# Sharp changes in tobacco products affordability and the dynamics of smoking prevalence in various social and income groups in Ukraine in 2008–2012

**DOI:** 10.1186/1617-9625-11-21

**Published:** 2013-10-18

**Authors:** Konstantin Krasovsky

**Affiliations:** 1 Institute for Strategic Research of the Ministry of Health of Ukraine, Kiev, Ukraine

## Abstract

**Background:**

To curb the tobacco epidemic, successful implementation of tobacco control measures should take into account how specific demographic groups react to particular policies. In 2005–2010, Ukraine experienced a sharp decline in smoking prevalence. In 2008–2010, several excise tax hikes combined with the economic recession resulted in a sharp reduction of tobacco product affordability, but in 2011–2012 tax increases were rather moderate. The aim of the current research was to investigate how smoking prevalence in various gender, social and income groups in Ukraine changed in response to differing tobacco taxation policies in 2008–2012.

**Methods:**

The State Statistics Service of Ukraine annual household surveys among the population aged 12 years and older, which include questions about smoking, were used. The aggregate data from the annual household surveys datasets of 2008–2012 were analyzed.

**Results:**

The decline in general smoking prevalence was much steeper in 2008–2010 – 3.2 percentage points in two years, while in two subsequent years it constituted only 0.6 percentage points. Smoking prevalence declined in all age, social, and income groups in 2008–2010. However, in 2011–2012 smoking prevalence continued to decline mainly among young and poor people, while some older and more affluent smokers apparently relapsed to smoking.

**Conclusions:**

Short-term and long-term price responsiveness of tobacco demand by socioeconomic status of population groups in low--and middle--income countries like Ukraine could be rather different for poor and more affluent people. Tobacco excise tax hikes have great potential in reducing smoking prevalence, especially in young and less affluent people, however they should also be supported by effective and available smoking cessation services.

## Background

To curb the tobacco epidemic, successful implementation of tobacco control measures should take into account how specific demographic groups react to particular policies. Experience in wealthy countries has shown that specific groups of the population characterized by a high prevalence of smoking may become resistant to the tobacco control measures shown to be otherwise highly effective. In this study, we consider data from Ukraine, which represents an interesting case study, having performed a rather intensive application of several tobacco control policies over a short period.

The WHO Framework Convention on Tobacco Control recognizes that price and tax measures are effective and important means of reducing tobacco consumption in various segments of the population, in particular in young people. Despite the public health rationale for increasing tobacco taxes to reduce tobacco use and its health and economic consequences, some authors dispute the social benefits of this intervention [1]. Among other arguments, opponents of higher tobacco taxes point out the negative distributional impact of higher tobacco taxes on the poor [1]. It was noted that to determine whether tax increases enhance the burden on the poor, it is important to know how the less and the more affluent groups will change their consumption due to increase in prices.

According to the WHO Reports on the global tobacco epidemic, between the Second (survey data collected in 2006 or earlier) [2] and the Third Reports (survey data collected in 2009 or earlier) [3], Ukraine has demonstrated one of the fastest declines in smoking prevalence in the world: age and sex standardized current tobacco smoking prevalence declined from 45% to 32%. According to the national reports, daily smoking prevalence in Ukraine decreased from 37.2% in 2005 to 25.5% in 2010 [3]. Ukraine has followed best international tobacco control practices, but the success has been achieved without governmental funding for tobacco control activities. Ukraine has almost not used those strategies which require even moderate national resources like quit lines or other cessation services [4]. The decline in smoking prevalence hence potentially resulted from the tobacco control legislation first adopted in 2005 and amended later, which included extension of smoke free policies; step-by-step tobacco advertising bans; large health warnings and other measures; however, most of these policies were implemented between 2005 and 2007.

The time between 2008 and 2012 is of specific interest for our study, as tobacco taxation dominated over other tobacco control policies in Ukraine those years. Since late 2008, several excise tax hikes were implemented [1]. In 2008–2010 average excise rate per pack of cigarettes increased six-fold: from 0.5 Hryvnas to 3.0 Hryvnas, while in 2011 the rate was increased by 7% and in 2012 – by 15%.

Concurrently with the most tangible tax increases, Ukraine experienced severe economic recession: in 2009, the Gross National Product (GNP) declined by 14.8%. The recession had strong impact on products affordability, including tobacco products.

Lower disposable income among the less affluent people would suggest that they are more sensitive to changes in prices and taxes compared to more affluent populations. In that case, tax increases would be progressive and help the poor to reduce their tobacco tax expenditures. However, the existing evidence is mixed and it has been suggested [1] that putative tobacco tax regressivity becomes the subject of further scrutiny. Thus the aim of this study was to explore how different gender, age and income groups reacted to the changes of tobacco products affordability. To achieve this goal we considered annually collected panel data presented by the national statistics agency in the aggregated form.

Smoking prevalence data were studied in the context of the sharp tobacco tax increase implemented only since late 2008 [5], hence smoking prevalence data of the household surveys in 2008–2012 was assessed.

## Methods

Since 2000, the State Statistics Service of Ukraine has been conducting annual household surveys among the population aged 12 years and older the “Population’s Self- perceived Health Status and Availability of Selected Types of Medical Aid” (sic). Each year more than 10,000 households take part in these surveys, which are conducted annually in October; details of the surveys have been presented in the reports [6-10]. The survey includes questions on many issues including smoking. Being a panel survey, it bears all the limitations of this type of surveys with expectable generalizability issues, as more affluent and mobile families often refuse to participate in the household survey. However, the sampling methods and the questions in the household survey have not undergone any changes over recent years and the survey results can be used to track recent trends. This data is in fact the best needed data for the purposes of our analysis and the only data available in Ukraine with consistent annual measurements.

The household surveys datasets were not available, so the aggregate data published in the reports were analyzed. When age groups were recomposed, their weights were taken into account. The question on smoking in the household survey is presented in a way that only daily smokers respond affirmatively, so in the below text “smoking prevalence” stands for only “daily smoking prevalence”. The respondents were lumped into 10 decile income groups. As these 10 groups were rather small for our analysis, we composed 5 income groups (quintiles), by uniting groups 1 and 2, 3 and 4 and so on.

Economic data on tobacco taxes, prices and revenues from the State Statistics Service database [11] were used to divide the time span into periods of differing tobacco affordability changes.

## Results

According to the State Statistics Service reports [6-10] daily smoking prevalence among the population 12 years old and over in 2008–2012 decreased from 25.6% to 21.8% or by 3.8 percentage points in four years. The total number of smokers in Ukraine declined from 10,069,000 in 2008 to 8,354,900 in 2012, a decline of 17%.

Data on smoking prevalence in various age groups of urban and rural population are presented in Table 1. Smoking prevalence among males is much higher than among females, especially in rural areas.

**Table 1:** Smoking prevalence in various age groups in Ukraine (%)

**Year**	**Smoking prevalence for population aged 12 years old and over (%)**	**Teenagers**	**Men, aged**			**Women, aged**		
		**14-17 years old**	**18–29**	**30–59**	**60 and older**	**18–29**	**30–54**	**55 and older**
**2008**	**25.6**	**5.7**	**53.3**	**59.9**	**32.6**	**13.3**	**9.2**	**1.6**
urban	26.7	5.5	52	59.6	31.3	16.4	11.9	2.3
*rural*	*23.4*	*7.2*	*57*	*60.5*	*34.7*	*4.2*	*2.8*	*0.5*
**2009**	**23.5**	**3.6**	**48.9**	**55.7**	**29.4**	**10.3**	**8.7**	**1.3**
urban	24.8	4.6	50.2	55.9	29.2	12.7	11.5	1.5
*rural*	*20.6*	*2.1*	*45.6*	*55.3*	*29.8*	*3.6*	*2.1*	*0.8*
**2010**	**22.4**	**4.4**	**45.8**	**52.4**	**30.8**	**9.6**	**8.2**	**1.4**
urban	24.0	4.5	47.4	53.2	32.8	11.8	11.0	2.1
*rural*	*19.1*	*4.4*	*41.9*	*50.7*	*27.4*	*3.0*	*1.8*	*0.1*
**2011**	**22.3**	**2.8**	**46.9**	**51.7**	**28.8**	**8.5**	**9**	**1.4**
urban	23.6	3.3	49.1	51.3	29.9	10.5	11.8	1.9
*rural*	*19.5*	*2.0*	*41.3*	*52.3*	*26.7*	*2.3*	*2.7*	*0.5*
**2012**	**21.8**	**1.9**	**42.6**	**51.7**	**26.7**	**7.3**	**9.4**	**1.8**
urban	*22.5*	1.7	43.0	50.7	25.1	8.9	12.0	2.6
*rural*	*20.4*	*2.3*	*41.7*	*53.7*	*29.5*	*2.5*	*3.3*	*0.3*
Rates of smoking prevalence decline in 2008–2012, prevalence ratio								
**Total**	**0.852**	**0.31**	**0.799**	**0.863**	**0.819**	**0.549**	**1.022**	**1.125**
urban	0.843	0.30	0.827	0.851	0.802	0.543	1.008	1.130
*rural*	*0.872*	*0.32*	*0.732*	*0.888*	*0.850*	*0.595*	*1.179*	*0.600*

**Figure 1:**
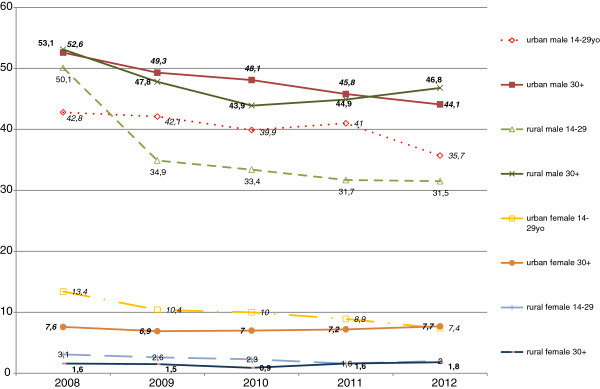
Smoking prevalence trends in different social groups in Ukraine in 2008–2012.

Rates of daily smoking prevalence declined in 2008–2012 were different in various age and gender groups (Table 1). The largest decline was among teenagers – smoking prevalence decreased three-fold. For males there were no big differences in the steepness of the decline by age group. Among females, we observed another trend: a sharp decline in prevalence among young women (below 30 years old) along with small increase of smoking among older women. Time trends of smoking prevalence in 2008–2012 for two large age groups: the “young” (14–29 years old) and “older” (30 years old and more) ones are presented on Figure 1. Smoking prevalence for young urban men is about 10 percentage points lower than for old urban men and the smoking decline in both groups in 2008–2012 was almost parallel and consistent. For rural men the decline time trends are different: the decline in 2008–2010 was steeper, than for urban men, but then smoking prevalence among older rural men increased in 2010–2011. For young rural men smoking prevalence continued to decline in 2011–2012, but much slower. Since 2009, the smoking prevalence for young rural men is more than 10 percentage points lower than for older urban men and it is lower than for young urban men.

Smoking prevalence greatly declined in young and older urban women in 2009, but then it continued to decline in young women, but increased in older urban women. While in 2008 smoking prevalence was much higher among young women, eventually in 2012 the prevalence rates became similar for old and young urban women. The smoking levels for rural young and old women also became similar in 2012, while in 2008 young rural women had twice higher smoking prevalence. Both for urban and rural old women some increases in smoking prevalence in 2010–2012 were observed.

**Figure 2:**
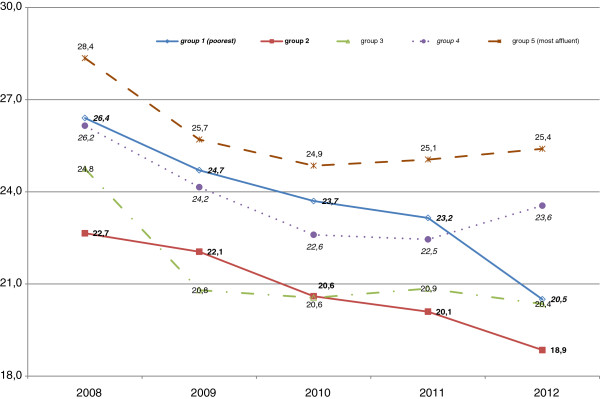
Smoking prevalence trends in different income groups in Ukraine in 2008–2012.

When smoking prevalence is compared by income groups, no direct relationship is seen between smoking rates and income as both the most and the least affluent groups have the highest smoking rates (Figure 2). In 2008–2010, the smoking rates declined almost parallel in all income groups. However, in 2010–2012, time trends for the income groups were different: smoking prevalence continued to decline in the two poorer groups, was stable in the middle group and in the two most affluent groups a slight increase was observed.

Overall, there were two different periods in the process of smoking prevalence change in Ukraine: 1) in 2008–2010 smoking prevalence declined in all age, gender, social and income groups and the overall decline was rather fast. 2) In 2010–2012, the smoking prevalence declined mainly among young and less affluent people, while it has slightly increased in some groups: older women, older rural men and more affluent groups. Overall, smoking prevalence still declined, but much slower than in previous two years. Decline was much steeper in 2008–2010 – 3.2 percentage points, while in two subsequent years it constituted only 0.6 percentage points.

## Discussion

Combined effects of the economic recession and the excise increase in 2009–2010 in Ukraine made cigarettes less affordable for most smokers and some of them tried to quit. In Ukraine from September 2008 to January 2011, the average excise per cigarette pack increased more than 6-fold. Over two years (2009–2010), the price index for tobacco products was 224%, while the general CPI (inflation) was 123%. The real (inflation adjusted) tobacco price increase was 83% (224/123=183).

It should be also taken into account that Ukraine experienced severe economic recession: in 2009, the Gross National Product (GNP) declined by 14.8%. In 2010, the GNP increased only by 4.1%. Hence the real affordability of cigarettes decreased two-fold (1.83 * 1.148 / 1.041= 2.02) in a short period of time. In 2011–2012, the excise increases were rather moderate and the real price increased only by 7% in two years. GNP increased by 5.2% in 2011 and by 0.2% in 2012, so the affordability of tobacco products remained stable in 2011–2012. Experience of other countries revealed that changes in tobacco products affordability is a key factor of tobacco consumption changes [12].

Other tobacco control policies (smoke-free places, tobacco advertising ban, health warnings, media campaigns) were gradually implemented in Ukraine since 2005 [4], but these policies usually have long-term effect and they potentially discourage smoking initiation more than they encourage quitting. It should be noted that new health warnings (text only warnings on 30% of front and back sides of the packs) were introduced in late 2006 [13]. Smoke-free policies were also introduced in 2006 [3]. So these kinds of tobacco control policies may not have had a significant impact on smoking prevalence in the period under consideration (2008–2012). Research conducted in such countries as USA, UK, Canada, Bangladesh, China, and Indonesia has indicated that smoking prevalence among men and women in lower socioeconomic groups is more responsive to the changes in cigarette prices [1]; however, in countries such as Egypt, Bulgaria, and Turkey the evidence is mixed [1]. In Ukraine, we see little difference in response to sharp price increase among different SES groups in short-term perspective, while in long-term perspective tobacco tax hikes have higher impact on smoking prevalence rates among younger and poorer people.

A recent study conducted in Australia [14] indicated that the tobacco tax increase was associated with a short-term increase in the rate of smoking cessation. In Germany, 4%–8% of smokers reported that the tax increases had prompted them to quit, with an increased likelihood of cessation efforts associated with a greater price increase [15]. In California, a significantly greater proportion of smokers reported quit attempts in the months immediately following the cigarette price increase; however, a significant increase in abstinence was only observed during the first four months after the price increase [16]. It is well known that approximately 90% of smokers who attempt quitting relapse within 6 months [17].

Policies that reduce tobacco products affordability have more obvious short-term effects and both discourage smoking initiation and encourage quitting. However, many smokers who tried to quit when cigarettes became much less affordable, may have relapsed, especially as smoking cessation services are hardly available, as in Ukraine [4].

Earlier research in Ukraine [18] demonstrated that in 2001, price elasticity of demand was much lower among people with high income. Among people with low and middle income, price elasticity of demand was -0.7 among adolescents (14–17 years old), -0.4 among young people (18–29 years old) and -0.3 among people above 30 years old [18], which is consistent with the results of the current study.

Similarly to our results, in India rural price elasticities for tobacco products were higher than urban ones, indicating that rural consumers are more price responsive [19]. In Ukraine, young rural men were the most responsive to the changes in cigarette affordability. It should be taken into account that “young” and “older” populations of 2008 and 2012 are somewhat different: people aged 25–29 years in 2008 were “young” (below 30), but in 2012 the same people became “older” (over 30) and actually they replaced other “older” people who died over these years. This cohort effect could have an impact on female smoking prevalence as women of 25–29 years old in 2008 had much higher smoking prevalence than women in older age groups. This phenomenon may partly explain the increase of smoking prevalence among “older” women in 2010–2012.

In general, the great reduction in tobacco products affordability in Ukraine in 2009–2010 had higher and more long-term impact on smoking prevalence among young and poor population groups. Recent research revealed that evidence on the variation of price responsiveness of tobacco demand by socioeconomic status of population groups is mixed in low--and middle— income countries [1]. The poorest people were not necessarily the most sensitive to tobacco price changes in these countries. Nevertheless, the results from Ukraine show that short-term and long-term price sensitivity could be rather different between less and more affluent people.

The study revealed that tobacco tax increases in Ukraine, as in many other countries, occurred to be progressive as they had higher impact on smoking prevalence among poor and young people. Tobacco excise revenues in Ukraine increased from 3.5 billion Ukrainian Hryvnas in 2008 to 16.6 billion Ukrainian Hryvnas (more than 2 billion US dollars) in 2012. Some part of the additional governmental revenues should be used for the establishment of smoking cessation services in Ukraine.

## Conclusions

Combined effect of excise tax hikes and the economic recession in Ukraine resulted in large reduction of tobacco product affordability in 2009–2010. This potentially caused the decline in smoking prevalence in all age, social and income groups.

However, this decline in affordability had long-term effect mainly on younger and poorer people, while some older and more affluent smokers apparently relapsed to smoking in 2011–2012, when tobacco product affordability was stable.

Tobacco tax hikes have more profound and long-term impact on smoking prevalence reduction among young and poor people.

Tobacco excise tax hikes have great potential for smoking prevalence reduction but the hikes are to be continued and they should be supported by effective and available smoking cessation services.

## Competing interests

The author declares that he has no competing interests.
